# Case of Strangulated Femoral Hernia

**Published:** 1855-02

**Authors:** 


Prof. Willard Parker, in the N. Y. Med. Times, reports a case of strangulated femoral hernia containing the ovary and fallopian tube of the right side. The fimbriated extremity of the tube was removed during the operation, supposing it to be a product of some previous inflammation. On dragging upon the ligature placed above the point of division the ovary appeared. It was replaced and the patient recovered without accident.

Another novel surgical operation is reported in the same journal by Dr. A. V. Williams. A man suffering from retention of urine, during a violent effort at micturition, felt something "snap," the desire to urinate subsided, and great pain in the belly supervened. A diagnosis was made of rupture of the bladder; Prof. Parker consulting.

As the only chance of life, an incision was made above the symphysis pubis, perpendicularly. On dividing the tendon in the linea alba, a copious flow of urine followed. The bladder was contracted behind the pubic. It was drawn up, punctured, and the finger introduced. The urethral orifice could not be felt. A sound was introduced *per urethram*, and felt pushing against the anterior wall of the bladder, which was divided to allow it to enter. The wound was brought together by a single suture, and a catheter inserted in the urethra. After a perilous illness from peritonitis, the patient recovered. Under the desperate circumstances such an operation was probably justifiable, but the principal wonder is in the recovery. H.

